# An Exploratory‐Descriptive Qualitative Study on Accessing Eye Health Services at the Community Level in the Gamo and Gofa Zones, Southern Ethiopia

**DOI:** 10.1002/hsr2.70272

**Published:** 2024-12-17

**Authors:** Tsegaye Alemu, Dawit Seyum, Mulate Gebre, Alemayehu Sisay

**Affiliations:** ^1^ School of Public Health, College of Medicine and Health Sciences Hawassa University Hawassa Ethiopia; ^2^ Orbis International Ethiopia Addis Ababa Ethiopia

**Keywords:** access, community level, eye health, obstacles, southern Ethiopia, women

## Abstract

**Background and Aims:**

Access to eye health care, which has a substantial influence on quality of life, is crucial for preventing and treating vision impairments. The determinants of access to eye health care in Ethiopia, especially in the Gamo and Gofa Zones in southern Ethiopia, are not well known. Therefore, this study aimed to identify barriers to accessing eye health services at the community level.

**Methods:**

A community‐based, exploratory descriptive qualitative study was conducted among adult women and men residing in two zones from June 2 to July 10, 2023. A purposive sampling technique was utilized to collect qualitative data. In‐depth interviews and FGDs were included among the purposefully selected study participants. During the interviews and discussions, digital audio recording was carried out. The data were analysed via the inductive thematic analysis technique and direct quotations from participants' opinions. The respective themes and subthemes provided a detailed overview of the findings, and the Atlas software was used to support the analysis.

**Findings:**

Nine FGDs were conducted among 90 participants. Moreover, a total of 26 in‐depth interview participants were included. The mean (±SD) age of the participants in the in‐depth interviews was 32.96 ± 4.8 years. The most commonly cited barriers (1) poor awareness and misperception, financial constraints and community‐based health insurance implementation gaps; (2) lack of social support; (3) lack of healthcare accessibility, shortage of basic medical equipment; (4) cultural eye care practices and (5) poor road access, rough topography, and difficulty reaching areas, which are identified as obstacles for accessing eye health services.

**Conclusion:**

This study identified pertinent barriers that hinder access to eye health care in the study area. Therefore, tailored strategies are needed to tackle the challenges. This approach is helpful for the local context and offers actionable insights for health policymakers and program designers.

## Summary

- Visual impairment and blindness are the most common in LMICs.
- Poor awareness and misperception, financial constraints, lack of social support, lack of healthcare accessibility, cultural eye care practices and poor road access make eye care services inaccessible.
- Improving accessibility (improving infrastructure and transportation), increasing eye care service availability, enhancing community health education and promotion to improve awareness of eye health diseases, and strengthening community‐based insurance schemes are key to tackling eye health barriers.

## 1 Introduction

In 2019, at least two billion cases of visual impairments were reported worldwide. Of these, at least one billion cases might have been prevented or are now unresolved [1]. Furthermore, up to 90% of affected individuals are located in countries with low‐ and middle‐income countries (LMICs). Fortunately, visual impairment may be avoided or corrected in more than 90% of individuals by existing and inexpensive interventions [2].

The existing evidence has shown that the lack of accessibility, cost of eye care treatment, and lack of availability are the primary factors contributing to the widespread occurrence of visual impairment, particularly in African countries [3]. Vision impairment severely affects people throughout their entire life. Timely access to top‐tier ophthalmic treatment may effectively decrease avoidable visual impairment. Moreover, visual impairment places a significant financial burden on the entire world [4]. Consequently, global eye health experts developed and stated three goals for Vision 2020, which sought to eliminate avoidable blindness. The first is the full application of the cataract and trachoma SAFE strategies. The second is to concentrate on enhancing human capital in emerging nations such as Ethiopia. Finally, significant eye health infrastructure investments need to be made to reduce eye health care access problems [5]. In 2022, the WHO reported that special efforts and strategies are needed to improve eye health access and affordability among disadvantaged populations in developing nations [1]. Therefore, providing and promoting universal eye health services is crucial for enhancing access to eye care services for most impoverished communities [6]. The existing evidence shows that maintaining good eye health is crucial for attaining the sustainable development goals (SDGs) [2, 7, 8].

The United Nations (UN) has made efforts to strengthen the eye healthcare system, and as a result, the Centre for Disease Control launched eye health care interventions, which are part of the national structure for preventing blindness and visual impairment. To enhance national eye care services, efforts have focused on addressing disparities or inequities in eye care [9]. Therefore, focusing more on reducing inequities among high‐risk populations is critical in the endeavour to lower the risk of visual impairment and loss [9, 10].

Ethiopia's eye health policies and programs reflect a comprehensive approach to addressing preventable blindness and improving access to eye care services. The government of Ethiopia signed and launched the VISION 2020 initiative in September 2002 [9], with support from this initiative; the Federal Ministry of Health (FMOH) has developed National 5‐year Eye Strategic Plans (2016–2020) in line with V2020 activities, which aim to address blindness and visual impairment [10]. Additionally, the government has taken steps to reduce preventable blindness by enhancing eye health access through the development and implementation of various eye health plans, such as the Health Sector Development Plan (HSTP) I [11] and the Health Sector Transformation Plan II [12]. Pertinent initiatives such as the National Cataract Surgery Program [13], school‐based vision screening programs [14] and the Trachoma Elimination Program have been initiated [15]. These policies are aligned with both national health priorities and global eye health initiatives, and they aim to increase the country's capacity to address eye health issues across urban and rural areas.

In addition, people of southern national nationality (SNNPs) reside in dispersed geographical locations, which may affect eye health delivery and access. Governments and partners have been exerting enormous efforts to eliminate avoidable vision impairment. However, there is little research on access to eye care in this area. Therefore, this study aimed to identify barriers facing vulnerable groups and suggest solutions to improve eye care delivery systems to better suit their requirements.

## 2 Methods and Materials

### 2.1 Study Design and Study Period

A community‐based, exploratory, descriptive, qualitative study design was employed to achieve the research objectives. This study was carried out from June 2 to July 10, 2023. The authors were utilized Consolidated Criteria for Reporting Qualitative Studies (COREQ)(S1).

### 2.2 Study Settings

This research was conducted in two South Nation Nationalities People Region (SNNPR) zones, namely, the Gamo Zone, Dita District, Gofa Zone, and Ubadebretsahay District. The Ubadebretsahay district is 555 km from Addis Ababa, the capital of the nation, and 273 km from the regional capital city of Hawassa. Agriculture and livestock production are the two primary economic sectors in the area. According to the Central Statistics Agency (CSA) population projection, 98,314 people live there, 48,174 males, and 50,140 women. The district has three health centres but no hospitals but two health facilities that provide eye care as well [16]. Dita District: This district is the second district where the study was conducted. It is located 375 km from the regional city of Hawassa and 563 km from Ethiopia's capital, Addis Ababa. According to the Central Statistical Agency (CSA) population projection, 121,247 people live there. Currently, five health centres provide primary eye care services [17].

There are two secondary eye care (SEC) facilities in zones: a two ophthalmologist, a four ophthalmic nurse, a five ophotometerist for human resources, a cataract surgery facility implemented at both SECs and a spectacle production centre at 01 SECs. In addition, there was community‐based health insurance (CBHI) and 70%–80% health insurance coverage in both study areas.

### 2.3 Study Participation and Recruitment Procedure

Adult women and men who were 40 years or older and who had eye diseases were purposefully selected for inclusion in this study during focused group discussions (FGD).

Health care professionals who had direct live experience with eye care programs, such as eye care professionals, integrated eye care workers, district NTD focal regional health bureau NTD focal, regional health bureau eye care focal, health extension workers, district health office heads and others, were included in the in‐depth interviews.

**Exclusion criteria:**

- The authors used the following criteria to exclude study participants:
- Individuals not residing in the Gamo and Gofa Zones were excluded.
- Individuals under 18 years of age were excluded to mitigate ethical issues.
- Participants with profound visual or cognitive impairment that obstructed communication.
- Individuals who declined to participate or who failed to provide informed consent were excluded from the research.

### 2.4 Sample Size Determination

The sample size in qualitative studies is often determined by the principle of data saturation—the point at which no new information or themes are observed in the data. For this type of study, particularly an exploratory‐descriptive qualitative study, sample size determination depends on several factors, such as an age group ≧ 40 years for FGD, sex (male & female), socioeconomic strata (lower, medium, & high) and various professional categories included to represent the population.

Therefore, a sufficient sample size is needed to explore and describe the participants' opinions fully and address the research question at hand. Consequently, we included 09FGD (participants who had eye health problems women and adults aged above 40 years) and 26 in‐depth interviews (participants such as HEWs, health centre directors, integrated eye care workers, district NTD focal, Zonal NTD focal, optometrists, ophthalmologists, and regional eye care focal) participants who were purposefully selected and included in the study. The data were collected until information saturation was reached.

### 2.5 Sampling Technique

A purposive sampling technique was utilized to collect qualitative data. FGDs and key informant interviews were considered to meet the research objectives.

Key informant interviews were carried out among purposefully selected health care experts, such as health extension workers, health centre directors, district health office heads, district neglected tropical disease focal staff, zonal health department neglected tropical disease focal staff, regional health bureau eye care experts, secondary eye care unit professionals such as ophthalmologists, ophthalmic nurses, integrated eye care workers, implementing partners and others who had adequate knowledge or expertise in the eye health programme in the SNNPR. In addition, FGDs were considered among female and male kebele residents (such as those who had eye health problems and were included in the discussions).

### 2.6 Data Collection Procedure

Following a review of the pertinent literature, interviews and discussion guides were created. For data collection, initially, the instrument was prepared in the English language, and then the English version was translated into the local language (Gamogna for FGD & Amharic language for in‐depth interviews). All the authors reviewed and critically reviewed the interview and discussion guides, and modifications were made accordingly. Before actual data collection, the developed interview and discussion guides were pretested at the Gofa Zone via six in‐depth interviews with health professionals who had experience providing eye health services and two FGD participants who were not involved in this particular study. Only a few modifications were carried out after the pre‐test was conducted. The actual data collection was carried out face‐to‐face in the field by a lead researcher and three experienced research assistants. During the interviews and discussions, participant privacy was maintained. To fully investigate the concerns, the participants were asked probing questions during the interviews. After everyone's permission was obtained, all the interviews and discussions were captured on a digital audio recorder. The average time taken for key informant in‐depth interviews and FGD discussions was 30–60 min.

### 2.7 Operational Definitions

Barriers to accessing eye health refer to the factors that prevent individuals from receiving the appropriate eye care treatments/services they need [18, 19]. These barriers can be categorized into several types: geographical barriers, financial barriers, sociocultural barriers, structural and organizational barriers, and technological barriers.

### 2.8 Data Quality Assurance

In this study, the trustworthiness of the qualitative data was maintained through credibility, transferability, debriefing, dependability and other criteria. To ensure study credibility, we first utilized the KII and FGD guidelines, which were reviewed by Orbis International Experts and Hawassa University professors. Before actual data collection, the instruments (discussion and interview guides) were pretested via six in‐depth interviews with participants who were health professionals who had experience with eye health service provision and eye health program management and two FGD participants who had an eye health problem. To obtain a greater range of perspectives from different participants, FGD participants were chosen on the basis of their age, sex, and eye health problems (diseases), and in‐depth interview participants were selected on the basis of their experience with eye health problems. In addition, the lead researcher and research assistant were also native speakers of the district language. This is crucial to lowering the likelihood of misunderstandings during the research and ensuring the validity of the conclusions.

For the second method, we used peer debriefing, which was carried out by qualitative experts from Hawassa University staff. This methodology focused primarily on the coding and analysis of qualitative data. The final tactic involved prolonged engagement throughout data collection in the field. The lead researcher and research assistant spent much of their time in the field and observed the points raised during the interviews and discussions, which helped us understand the actual scenario.

The third method, the study setting and the study participant characteristics, are precisely described in the methods section to guarantee the general transferability of the research procedure. Moreover, between each interview question, probing techniques were used, such as asking more questions to learn more about the respondent's perspective. The data collectors took written field notes and used a digital audio recorder to capture the interviews.

### 2.9 Qualitative Data Management

In this study, qualitative data analysis was started at data collection sites by considering field notes, voice recordings and debriefing during the data collection phase. Then, the recordings were labelled correctly along with their field notes, and the audio was translated verbatim by the lead researchers of the study. Before coding began, the transcripts were reread several times by researchers and assistants to build a consensus (intercoder agreement) via ATLAS. ti 23; then, the codes were edited and deleted as appropriate. Then, each code report was generated, cleaned, and prepared for synthesis after the coding was finished. Then, we synthesized each code, where necessary, removed or merged any codes that no longer appeared and classified all remaining codes into these domains. The quotations in the results section are identified only by source, such as FGD/KII, location, and informant group, where applicable.

## 3 Findings

### 3.1 Socio‐Demographic Characteristics of the KII and FGD Participants

Table 1 provides a list of participants in the FGD from different sites and zones. The FGD participants are grouped on the basis of their respective *kebeles* and zones. The data included the number of male (M) and female (F) participants at each site. Nine FGDs were conducted with a total of 90 participants (women = 52 and men = 38).

**Table 1:** Socio‐demographic characteristics of FGD participants in the SNNPR, 2023.

No	Site	Zone	Sex		No. of participants	Age range	Economic status
			**M**	**F**			
1	Shele Kebele	Gofa	3	5	8	40–90	Farmer
2	Ubyela Kebele	Gofa	4	6	10	45–80	Farmer
3	Giyassa Kebele	Gamo	5	6	11	43–89	Farmer
4	Bala Kebel	Gofa	5	5	10	44–85	Farmer
5	Beto Kebel	Gofa	5	7	12	47–98	Farmer
6	Mehal Beto	Gofa	3	6	9	40–95	Farmer
7	Zaba Kebel	Gofa	4	5	9	42–80	Farmer
8	Donne Kebele	Gamo	4	6	10	42–76	Farmer
9	Dita Suluse	Gamo	5	6	11	46–70	Farmer

The KII participants (Table 2) were diverse groups of participants with different qualifications and experiences. They held positions such as IECW, optometrist, ophthalmologist, ZHD head, WoHo head, HC head, HEW head, eye care focal, integrated eye care worker, NTD focal, and DPHP coordinator, with less than a year to 16 years of experience. The mean age (±SD) of the KII participants was 32.96 ± 4.8 years, and 34.6% (*n* = 9) of the participants were female. A total of 26 participants (9 females and 17 males) were included in the in‐depth interviews.

**Table 2:** Socio‐demographic characteristics of the KII participants in the SNNPR, 2023.

SN	Variable	Frequency	Percent
	Age (Mean ± SD)	32.96 ± 4.8	
	Experience (Mean + SD)	8.0 ± 5.0	
1	Sex		
	Female	9	34.6
	Male	17	65.4
2	Qualification		
	Diploma	11	42.3
	Degree	11	42.3
	Master and above	4	15.4
3	Position		
	DPHP Coordinator	2	7.7
	Eye focal	1	3.8
	Head	5	19.2
	HEW	13	50.0
	NTD Focal	1	3.8
	Ophthalmologist	2	7.7
	Optometrist	2	7.7

A total of 26 study participants were included in this study. Of these, 9 study participants were female, 7 held diplomas, 1 held a master's degree, and 1 had an ophthalmologist qualification. On the other hand, of the total study participants, 17 were male, 11 were male and had a bachelor's degree, 4 had a diploma, 1 had a master's degree, and 1 had training in ophthalmology.

In addition, 6 male participants were in the health facility head position, 6 participants were integrated eye care workers, 2 participants were in the optometrist position, 2 participants were in the disease prevention and health promotion position, and 1 participant was in the NTD focal position, whereas 6 female participants were in the health extension worker position, 1 female participant was an integrated eye care worker, and 1 female participant was in the ophthalmologist position.

The qualitative assessment revealed five main themes: individual‐, interpersonal‐, community‐, health system‐, and societal‐level factors. Each theme encompasses subcategories that shed light on various aspects. Under the individual‐level theme, subcategories include poor awareness, financial constraints and CBHI. At the interpersonal level, social support emerged as a significant factor. The health system level consists of three themes: health service accessibility, trained staff shortages and heavy workloads, and structural and functional factors. The societal‐level theme represents the fourth theme, and within it, we find three categories, namely, policy and governance, along with contextual factors and socioeconomic factors. Finally, the community‐level theme has two categories: cultural beliefs and malpractice (Table 3).

**Table 3:** Themes and subthemes that emerged from the data of 2023, the SNNPR, and Ethiopia.

Themes	Subthemes	Category
Individual	Poor awareness and misperception	Lack of eye knowledge, poor awareness about eye care
	Financial constraints and CBHI	Lack of finance to go eye care unit, lack of finance to transport eye care clinic
Interpersonal	Social support emerged	Older patients lack support to visit eye care unit, lack of family to brought them to eye care units,
Health system	Health service accessibility lack of equipment	Lack of eye care units near to the community, high topography to access eye care units, no functional eye services
	Trained staff shortage and workload	Lack of trained eye care professionals, poor skill of eye care professionals, not committed on eye care services provisions
	Structural and functional factors	Lack of eye care structure at community level, no functional integrated eye care workers
Societal level factors	Policy and governance	Poor eye care police implementation, no regular eye care screening program at community, no regulatory body on eye services
	Contextual factors	contextual barriers such as poor road access, rough topography, and hard‐to‐reach areas
	Socioeconomic factors	Lack of money to pay for eye care services, lack of money for eye care check ‐up, even for transportation
Community	Cultural beliefs & malpractice	Practicing traditional issues on eye rather than modern eye care medicine

#### 3.1.1 Themes: Individual Level

##### 3.1.1.1 Subtheme: Poor Awareness and Misperception

Poor awareness and misperceptions about the importance of seeking eye care services were highly evident, which highlights the prevailing problems in the community. The barriers under this category relate to associating the illness with age, being unaware of eye care services available in health facilities, and assuming that it is untreatable and unaware of the necessity of regular check‐ups. Poor awareness potentially leads to unaddressed eye health issues, as the respondents say.

An ophthalmologist working in a general hospital mentioned that people view eye issues as a natural part of ageing, considering it a grace of being elderly people. As a result, individuals tend to neglect professional eye care, assuming that problems are expected and unavoidable due to their age.

Most ophthalmology cases are seen as something that comes with age, so people take it as a grace of being elderly people, and people rarely seek care. Therefore, they do not seek care.

[KII, ophthalmologist]

A significant factor contributing to the lack of awareness of eye care services is the absence of knowledge regarding treatability. Many informants and discussants reported that people with poor eye conditions are not aware that their eye conditions can be effectively treated, leading to delayed or neglected care‐seeking behaviour.

Another crucial aspect is the lack of awareness about the possibility of treatment. Consequently, people often delay seeking care until treatment is no longer useful.

[KII, IECW]

In addition to the previously mentioned challenges, a prevailing misperception reported by the participants was the community belief that undergoing eye surgery can lead to blindness. This misconception contributes to people being hesitant or unwilling to seek eye care services. A head of a health facility explained saying…

There is a strong belief that once eye surgery is performed, blindness can occur, so many patients are not coming to the facility.

[KII, HC head]

The FGD participants also reaffirmed that “Community members prefer to use traditional healers and lack knowledge about the importance of early eye care screening and treatment of eye diseases*”* [FG3, P4].

Other FGD participants also noted the following:

Most of our community members were unaware of the availability of eye health services in nearby health facilities.

[FG7, P 6]

##### 3.1.1.2 Subthemes: Financial Constraints and CBHI

Most of the participants indicated that poverty and a lack of income sources pose significant obstacles to accessing eye care services. While certain medical costs related to eye care may be free, indirect expenses, including transportation, foods, and accommodations, were consistently identified as the main barriers preventing individuals from seeking and utilizing eye care services.

Poverty and the absence of stable income sources create formidable barriers for people accessing eye care services. Although some medical costs are waived, additional indirect expenses, such as transportation, food, and accommodations, heavily influences the decision to seek necessary eye care.

[KII, optometrist]

It was further reaffirmed by FGD *participants…we want to go and seek care but how can we afford the cost motorcycle and where will we stay* [FG4, P8].

According to the majority of participants, three consistently reported financial constraints are associated with the CBHI. The first issue pertains to CBHI members receiving free eye care services and diagnoses but facing difficulties in obtaining medication and reimbursement. The facility is expected to provide prescriptions, but they end up going to private pharmacies, which they claim cannot afford to purchase from private pharmacies.

CBHI enrolment is above 60% in the woreda, but drug shortages are critical; they are insured but not getting drugs…if you send them to private pharmacies, they can't, but it is meaningless to diagnose them if you don't treat.

[KII, NTD focal]

Notably, however, key informants from zonal‐level discussions reported that drug unavailability is related not only to eye care services but also to all other essential services. A zonal deputy reported….*it is not only eye care service, but all others also do not have the drugs despite being enrolled in CBHI* (KII, ZHD deputy).

The second scenario involves many families unable to afford CBHI payments due to the high number of destitute families. Although the WrHO covers the cost for a few destitute families, many others cannot afford the service. For example, they reported that single‐eye surgery costs between $14 and $20, not to mention additional expenses such as medication, transport, and follow‐up costs. It is unrealistic to expect people who fail to pay less than $10 for CBHI to be able to cover the expenses of a single‐eye surgery.

A 40‐year‐old mother from an FGD discussion mentioned that the reason for not going to a health facility was that she did not benefit from the WrHO offices’ health assistance. *…here there are many indigent families, but the woreda picks very few then how can I go if I don't have CBHI cards [I am not insured]* [FGD 3, P6].

The third scenario relates to the fact that the CBHI does not cover some vital eye care service costs, especially for eyeglasses. An optometrist with more than 3 years of experience said, *CBHI helped to obtain services but not spectacles* [KII, optometrist].

Notably, most informants reported that the CBHI has helped many people in seeking treatment despite all the odds compared with less enrolment.

#### 3.1.2 Themes: Interpersonal Level

##### 3.1.2.1 Subtheme: Social Support

The other category that emerged was lack of social support, which is a consistently reported barrier to accessing eye care services. The participants emphasized that certain vulnerable groups, such as preschool children and elders older than 40 years, who require assistance and accompaniment to health facilities, face challenges in seeking eye care. The absence of adequate social support for individuals with visual challenges hinders their ability to visit health facilities, often compelling them to remain at home instead of seeking necessary eye care services.

A health centre head concluded that vulnerable people usually stay at home.

Eye problems mostly affect preschool children and elderly individuals, who often need support and assistance to access health facilities. They are mostly overlooked and left at home rather than being taken to facilities.

[KII, HC head]

The inability to receive sufficient social support was further reaffirmed by other participants saying*…. Elderly people with eye conditions need someone to travel to health facilities, and the distance is not easy* [KII, NTD focal].

#### 3.1.3 Theme: Health System Level

##### 3.1.3.1 Subtheme: Healthcare Accessibility

The qualitative study findings indicate that residents of rural areas face significant challenges accessing eye care services because of the considerable distance to health facilities. The participants consistently reported that it takes 2–8 h on foot for a round trip to seek eye care services, making this a major barrier to accessing such services. None of the discussants from rural settings mentioned a travel time of less than 2 h to reach the nearest eye care facility.

A 52‐year‐old elderly individual described how far a nearby health centre could take up to 8 h for a round trip.

We go 3 to 4 h on foot to reach the health centre, so it takes 6 to 8 h to go and get back. Elderly people cannot come and obtain services.

[FGD1, P4].

The other access reported by the participants related to the fact that despite having insurance and the right to receive eye care services, few did not have skilled providers to deliver the service. A zonal health head reported that there is a high enrolment rate, but members do not benefit from the insurance agreement.

We have 85% CBHI enrolment, but the problem is that they do not receive eye care services despite being insured because there is no trained provider in eye care.

[KII, ZHD]

##### 3.1.3.2 Subtheme: Trained Staff Shortages and Workload

The other main issue identified is that the staff trained in the IECW face a heavy workload. They are expected to provide eye care services as an additional task rather than as their primary role. As a result, they continue to handle all their previous activities and plans alongside their new responsibility of providing eye care services.

The main problem is that we have a high workload; we are providing eye care services as an additional task, not as a main role, because we are still handling all other activities and plans as before and eye care services as an additional role.

[KII, IECW]

This finding was further reaffirmed by an optometrist from a hospital who reported a lack of focus and attention given to the eye unit by the administration.

The eye unit seems to receive less priority and support from the administration, leading to its inadequate functioning.

[KII, Optometrist]

Although a shortage of providers was consistently reported, the other concern reported by higher adolescents was related to the monitoring system of those trained to provide eye care.

Despite having numerous trained IECW professionals, none of them are actively working due to the absence of a clear monitoring system to track their activities and locations.

[KII, WrHO]

##### 3.1.3.3 Subtheme: Structural and Functional Factors

The other consistently reported barriers relate to the availability of basic equipment to deliver the service; they reported a lack of essential equipment, such as IOP measuring tools, torno meters, and minor sets, and the absence of skilled people to maintain nonfunctional equipment, along with supply shortages, which pose significant challenges in providing adequate eye care services.

We do not have equipment such as IOP measuring tools, and nobody fixes equipment that is not working. Additionally, there is no minor set available, and supply shortages present a challenge in providing eye care services, as we lack the basics.

[KII, Optometrist]

Apart from the challenges related to structure, another notable issue is functional‐related challenges among eye care service providers. Unlike other professionals, eye care providers do not receive adequate benefits or duty payments, leading to poor motivation among them.

Poor motivation from professionals’ side… no duty payment or benefit… eye care providers are excluded from benefits all in all.

[KII, Optometrist]

#### 3.1.4 Theme: Community Level

##### 3.1.4.1 Subtheme: Cultural Practices/Malpractices

Several malpractices are used for eye treatments according to participants, ranging from simple drops of plant extracts to the use of sharp metals to cut and pierce the eyes.

Although the participants did not perfectly align what to what treatment, few mentioned trachoma and cataract as conditions from which they use plants such as “Gololo and Moringa plant”.

A 45‐year‐old male discussant described saying…*in our village, people squeeze plants like Gololo and* Moringa plant *and then drop their water on the ill eyes* [FGD 5, P7].

Many participants reported that they mix a tetracycline tab with butter and apply it to the ill eye. An elderly reported saying…*What we do as a traditional treatment here is that TTC pill dusting is mixed with butter and Vaseline, which are then applied to the eye. The thing is that we drop those plant droplets (water) when our eye itches us; then, our eye will turn red, and we say it turned red, which is a sign of healing* [FGD 3, P2].

The other treatment reported relates to the use of goat milk…*A participant mentioned… we also use goat milk as treatment when our eyes get ill* [FGD2, P2].

The other malpractice noted relates to the local name called the *ZAMA*. According to the participants, the name *“ZAMA”* refers to a condition where the spider gets into the eyes or moves around, then cuts the eye surroundings *they believe DAFANA grows up…. and they fear that it leads to pus corneal ulcers, “nerve metekos”*.

*Sociocultural: In rare cases, individuals sometimes turn their eyelids upside down and bleed using sticks as a treatment when itches. Moreover, the eyelashes were removed for treatment*.

#### 3.1.5 Theme: Societal Level

##### 3.1.5.1 Subtheme: Policy and Governance

The other category that emerged relates to sole eye care tasks given to an NGO called ORBIS. According to the informants, neither the government nor eye care service providers work to take over the task; rather, they continuously expect support from nongovernmental organizations such as ORBIS.

The prevailing perception is that all eye‐related tasks require assistance from ORBIS, but this mindset needs to be changed. Health facilities seem unprepared to provide any services themselves, relying heavily on ORBIS for their expectations.

[KII, NTD focal]

##### 3.1.5.2 Subtheme: Contextual Factors

The other category that emerged that all participants reported related to contextual barriers such as poor road access, rough topography, and hard‐to‐reach areas, which are mentioned as deterrents *to accessing eye care services*. A zonal head described saying…. *There are places unimaginable to think of going to health facilities, no road access or transportation by any means and others* [KII, ZHD head].

Another participant further described that *during the rainy season, certain areas become nearly impossible to reach, with limited or no road access or transportation available, making it difficult for people to access health facilities in those remote and hard‐to‐reach areas* [KII, WrHO head].

##### 3.1.5.3 Subtheme: Socioeconomic Factors

The majority of FGDs and in‐depth interviews revealed inequity in accessing available eye healthcare services on the basis of age, gender and socioeconomic status.

In‐depth interviews with participants from the Sawela secondary eye health professional revealed the following:

*…*I am noticing that females suffer more from eye disease, but they are not visiting the eye care unit for different reasons (32‐year‐old optometrist).

However, another participant contradicted the aforementioned statement:

*…*I didn't see any difference in male and female eye care visits (35‐year‐old female ophthalmologist).

In addition, other in‐depth interview participants explained this:

…In our secondary eye facility, old people are not visiting the eye facility because they perceive that vision loss in old age is normal and acceptable when age increases (Ophthalmologist, 37 years old).

The FGD participants also noted that:

“Women, especially those with caregiving responsibilities in home, faced significant barriers to accessing care, such as social and cultural expectations, which often limited their ability to seek healthcare, which was seen as less of a priority than their familial duties”.

Another in‐depth interview participant described the following:

The majority of eye care visiting patients have a relatively better income and can pay for eye care services, whereas those patients who have a lower income category do not visit eye clinics regularly, and even if they visit, they cannot pay for eye care services because the woreda or other third party mostly covers their expenses (34‐year‐old optometrists).

## 4 Discussion

In the current study, we explored barriers to accessing eye health services at the community level in the Gamo and Gofa Zones, SNNPR, Ethiopia. In this study, we identified 5 themes and 10 subthemes that emerged from the gathered data. The five main themes are individual‐, interpersonal‐, community‐, health system‐, and societal‐level factors. Each theme encompasses subcategories that shed light on various aspects. Under the individual‐level theme, subcategories include poor awareness, financial constraints and CBHI. At the interpersonal level, social support emerged as a significant factor. The health system level consists of three themes: health service accessibility, training staff shortages and heavy workloads, and structural and functional factors. The societal‐level theme represents the fourth theme, and within it, we find three categories, namely, policy and governance, along with contextual factors and socioeconomic factors. Finally, the community‐level theme has two categories: cultural beliefs and malpractice, which emerged from the data.

In this study, poor awareness and misperception of the importance of seeking eye care services were highly evident, which highlights the prevailing problems in the community. The barriers under this category relate to associating the illness with age, being unaware of eye care services available in health facilities, and assuming that it is untreatable and unaware of the necessity of regular check‐ups. Poor awareness potentially leads to unaddressed eye health issues. This finding is supported by a study conducted by Benchage and colleagues (2021) in Pakistan on improving access to eye health services in rural communities, which revealed that low eye health awareness hinders eye health service access [20]. A systematic review of eye health barriers conducted by Piyasena et al. (2019) revealed that a lack of knowledge, attitudes, and awareness were identified as key barriers that affect eye health access [21]. Moreover, another systematic review was conducted by Alrasheed in 2021 in African countries to determine barriers to accessing paediatric eye care services, and the reviewed evidence demonstrated that lack of knowledge and attitudes were major barriers to accessing eye care [3]. A qualitative study conducted by Leamon and colleagues in the UK indicated that limited awareness of eye health and eye disease are barriers to accessing eye services [22, 23]. Other evidence from Iran also revealed that poor awareness is a major barrier to eye health service utilization [24].

Our study revealed that most participants indicated that poverty and a lack of income sources pose significant obstacles to accessing eye care services. While certain medical costs related to eye care may be free, indirect expenses, including transportation, foods, and accommodations, were consistently identified as the main barriers preventing individuals from seeking and utilizing eye care services. This finding is similar to the findings of a study in Mozambique Nampula, which indicated that low monthly income was significantly associated with barriers to accessing eye care. These factors hinder the utilization of eye care services, and specific intervention strategies are needed [19]. Another study performed in Hawassa, Ethiopia, revealed that having a family monthly income greater than or equal to $120 in Ethiopia was positively associated with the utilization of eye care services [25]. A similar study revealed that wealthy people were more likely to visit eye care facilities than were those with low wealth status [26].

Our study revealed that many families were unable to afford CBHI payments because of the high number of destitute families. Although the district covers the cost for a few destitute families, many others cannot afford the service. For example, they reported that single‐eye surgery costs between $14 and $20, not to mention additional expenses such as medication, transport, and follow‐up costs. It is unrealistic to expect people who fail to pay less than $10 for CBHI to be able to cover the expenses of a single‐eye surgery. This finding is similar to the existing evidence that demonstrated that a lack of health insurance was the most common barrier to utilizing the service (32.3%) [27, 28].

The present qualitative study revealed that a lack of social support is a consistently reported barrier to accessing eye care services. The participants emphasized those certain vulnerable groups, such as preschool children and elders older than 40 years, who require assistance and accompaniment to health facilities, face challenges in seeking eye care. The absence of adequate social support for individuals with visual challenges hinders their ability to visit health facilities, often compelling them to remain at home instead of seeking necessary eye care services. This finding is similar to that of a study conducted in southern India, which revealed that a lack of family support was the most common barrier to the use of cataract surgery. However, having social support from neighbours (28.6%), being a general health worker (20.2%) and having a previous history of cataract surgery (19.6%) are factors promoting the use of cataract surgery [29]. In addition, a study by Pakistain on barriers to eye care in a hard‐to‐reach population reported that social support limitations were barriers to eye health access in people who were hearing from communities [30]. A recent study revealed that there is insufficient focus on child eye health in key Ethiopian health policy documents and that children's eye health access in Ethiopia is limited. Therefore, policies, partnerships, collaboration and coordination of stakeholders are suggested to improve child eye health services [31]. Consequently, child cataract output is very low [32]. A systemic review report from African countries indicated that child eye care has many barriers to accessing paediatric eye care services [3]. Therefore, eye health promotion interventions substantially improve the eye health attitudes, knowledge, and practices of school children. In addition, the engagement of parents and teachers as change agents may further improve children's health [33].

Our qualitative study findings indicate that residents of rural areas face significant challenges accessing eye care services because of their considerable distance from health facilities. The participants consistently reported that it takes 2–8 h on foot for a round trip to seek eye care services, making this a major barrier to accessing such services. None of the discussants from rural settings mentioned a travel time of less than 2 h to reach the nearest eye care facility. This finding is comparable to that of a previous descriptive study, which revealed that barriers related to infrastructure, such as distance from the patient's home to a health facility, hinder eye health access [34, 35].

According to this study, all the participants cited contextual barriers such as poor road access, rough topography, and difficulty reaching areas that are mentioned as deterrents *to accessing eye care services*. A zonal NTD focal described saying…. *There are places unimaginable to think of going to health facilities, no road access or transportation by any means and others* [In depth, ZHD head]. This finding is comparable with previous evidence indicating that the absence of transportation was a crucial barrier to utilizing eye care [27]. Other studies have indicated that road problems, geographical challenges and transportation problems are the main factors that significantly impact barriers to accessing eye care [3, 30, 35, 36].

The other access barriers consistently reported by the participants were a shortage of trained eye care providers despite having insurance and the right to receive eye care services, and the majority did not have skilled providers available to deliver the service. Even if they are few in number but have a low workload and, unlike other professionals, eye care providers do not receive adequate benefits or duty payments, leading to poor motivation among them. These findings are consistent with a qualitative study conducted by Zaback et al. (2020) showing that a lack of access to eye care providers affects eye care access [37]. Another study conducted in Nigeria revealed that eye care workers were disproportionately distributed, which created a barrier to the acceptance of eye care services [38]. Similarly, there is insufficient clinically trained manpower to meet community eye care needs, which may affect the quality of service provision in sub‐Saharan Africa [39]. Similar evidence from Sri Lanka confirmed that the allocation of human resources (HR) was not based on population demands; three‐fourths of the eye care facilities are located in one province, and ophthalmologists are unevenly dispersed, with most remote needy communities lacking access [40]. Therefore, sub‐Saharan Africa, including Ethiopia, needs a strong emphasis on the development of the human resources for eye health care required to reduce preventable visual impairment [41].

The other consistently reported barriers relate to the availability of basic equipment to deliver the service; they reported a lack of essential equipment, such as IOP measuring tools, torno meters, and minor sets, and the absence of skilled people to maintain nonfunctional equipment, along with supply shortages, which pose significant challenges in providing adequate eye care services. This finding is in line with a study conducted in Nampula, Mozambique, where some key eye care service delivery equipment was lacking at primary and secondary health institutions, making it difficult for them to deliver services related to eye health [42]. In a similar study performed in Nigeria on the availability of eye care equipment, infrastructure, and primary eye care supplies, there was no discernible difference between health centres and health posts. Fewer than one‐third of the facilities had working torches, and the majority lacked visual acuity charts [43]. Therefore, to meet the global vision goal, every country should design unique strategies to confirm the equitable distribution of eye care equipment, eye professionals and infrastructure [44].

This study revealed that several cultural malpractices, ranging from simple drops of plant extracts to the use of sharp metals to cut and pierce the eye surroundings, are used for eye treatments. Although the participants did not perfectly align what to what treatment, few mentioned trachoma and cataract as conditions from which they use plants such as “Moringa plant and Gololo”. A 45‐year‐old male discussant described saying…*in our village, people squeeze plants like Gololo and Shiferaw and then drop their water on the ill eyes* [FGD 5, P7]. This finding is in line with a previous qualitative study conducted in Sri Lanka that indicated that sociocultural factors were barriers to accessing eye care [45]. An existing systematic review of African countries revealed that cultural factors (belief and perception) are key barriers to accessing eye health [3]. A study from Uganda and Zimbabwe revealed that plant products were the most often utilized type of traditional eye medicine (TEM) [46, 47]. Sub‐Saharan Africa (SSA), including Ethiopia, utilizes home‐based remedies and TEM as first‐line eye treatments for eye conditions and practices self‐prescribed ophthalmic medications to relieve ocular symptoms. TEM practices are deeply rooted in cultural beliefs and are often passed down through generations. In many rural communities in SSA, traditional healers are highly respected and consulted for various health issues, including eye conditions [48, 49, 50, 51]. Other evidence has indicated that there is a significant challenge in eye care access in SSA, which may require intervention [52].

Our findings revealed that the majority of FGDs and in‐depth interviews cited inequity in accessing available eye healthcare services on the basis of their age, gender and socioeconomic status. This finding is supported by documented evidence of inequality and inequity in eye health, which indicates that even when eye care services are free, they are sometimes inaccessible to those who live in rural areas or poverty [53]. Similarly, economic and gender inequalities are barriers to eye care utilization [19, 54]. Another qualitative study from Cambodia revealed that women were a vulnerable group; however, they did not access eye care services because of their lower socioeconomic backgrounds and because they resided in remote rural locations [55]. Similarly, existing evidence indicates that there are disparities in access to eye care services in terms of socioeconomic status, gender, age, and geographical location [56]. Therefore, every eye care professional should plan to offer eye health services of the same quality to everyone, regardless of their age, gender, wealth, ethnicity, place of residence, level of education, or level of disability [53].

## 5 Strengths and Limitations of the Study

We used multiple data sources (FGDs and in‐depth interviews) to respond to the research objectives. Moreover, adequate study participants were considered. However, this study has several limitations. The FGD study was conducted in Gamo and Gofa Zones, which are rural areas. Consequently, the findings may not be generalizable to other urban areas that may have different sociocultural characteristics. Moreover, this research offers a timely view of obstacles to timely access to eye health care. Changes in infrastructural development, economic situations, or healthcare regulations after the research might change some of the obstacles found.

## 6 Conclusion

In this study, the following obstacles were most commonly cited for eye care access: poor awareness, misconception and financial constraints and CBHI; social support, which has emerged as a significant factor; health service accessibility; training staff shortages and heavy workloads; structural and functional factors; policy and governance; socioeconomic factors; cultural beliefs; and malpractice. Therefore, researchers strongly suggest advocacy for high‐level eye health promotion to increase eye health care awareness, conduct regular discussions with communities that influence malpractice in eye health care, and provide eye health services for those who have difficulty reaching residents. Moreover, local governments need to address health facility infrastructure and lack road access and need to work on income generation activities to alleviate poverty.

## Author Contributions

Conceptualization: Tsegaye Alemu, Dawit Seyum, Mulate Gebre, and Alemayehu Sisay. Data collection: Tsegaye Alemu, Dawit Seyum, Mulate Gebre, and Alemayehu Sisay. Formal analysis: Tsegaye Alemu, Dawit Seyum, Mulate Gebre, and Alemayehu Sisay. Investigation: Tsegaye Alemu, Dawit Seyum, Mulate Gebre, and Alemayehu Sisay. Methodology: Tsegaye Alemu, Dawit Seyum, Mulate Gebre, and Alemayehu Sisay. Supervision: Dawit Seyum, Mulate Gebre, and Alemayehu Sisay. Writing–original research draft writing: Tsegaye Alemu, Dawit Seyum, Mulate Gebre, and Alemayehu Sisay. Review and editing: Tsegaye Alemu, Dawit Seyum, Mulate Gebre, and Alemayehu Sisay.

## Ethics Statement

The southern national nationality regional health bureaus of public health institutions granted ethical clearance (Ref number pw6‐19/9643 & date 23/06/2023). Moreover, SNNP RHB, Gamo ZHD, and Gofa ZHD also provided permission letters to carry out the study. All ethical principles were maintained strictly.

## Conflicts of Interest

The authors declare no conflicts of interest.

## Transparency Statement

The lead author Tsegaye Alemu affirms that this manuscript is an honest, accurate, and transparent account of the study being reported; that no important aspects of the study have been omitted; and that any discrepancies from the study as planned (and, if relevant, registered) have been explained.

## Data Availability

All the data reported in the manuscript are publicly available upon the official request of the principal investigator upon acceptance of the manuscript.
